# Inevitable Evolutionary Temporal Elements in Neural Processing: A Study Based on Evolutionary Simulations

**DOI:** 10.1371/journal.pone.0001863

**Published:** 2008-04-02

**Authors:** Uri Yerushalmi, Mina Teicher

**Affiliations:** The Leslie and Susan Gonda Interdisciplinary Brain Research Center, Bar-Ilan University, Ramat-Gan, Israel; Minnesota State University Mankato, United States of America

## Abstract

Recent studies have suggested that some neural computational mechanisms are based on the fine temporal structure of spiking activity. However, less effort has been devoted to investigating the evolutionary aspects of such mechanisms. In this paper we explore the issue of temporal neural computation from an evolutionary point of view, using a genetic simulation of the evolutionary development of neural systems. We evolve neural systems in an environment with selective pressure based on mate finding, and examine the temporal aspects of the evolved systems. In repeating evolutionary sessions, there was a significant increase during evolution in the mutual information between the evolved agent's temporal neural representation and the external environment. In ten different simulated evolutionary sessions, there was an increased effect of time -related neural ablations on the agents' fitness. These results suggest that in some fitness landscapes the emergence of temporal elements in neural computation is almost inevitable. Future research using similar evolutionary simulations may shed new light on various biological mechanisms.

## Introduction

Many current Neural Network models assume that all semantic information is contained in the spike rates of the neurons [1]. But there is also evidence that the fine temporal structure of the spiking activity may play a role [2].

Most recent research on time - dependent neural computation has focused on examining the computing power of temporal neural computation models [3] or on uncovering biological evidence that supports the claim of precise neural activity timing [4], [5]. However, from an evolutionary point of view, little is known about the circumstances that may have prompted the evolution of temporally based neural computing systems. One such circumstance could have been the need for a binding mechanism, as presented in [6], which posits a compositionality model where synfire chain waves [7] represent semantic atoms and synchronization of activity in different chains serves as a binding mechanism. Recently, it has been shown [8] through simulations that such a model is actually possible and is able to solve simple binding problems. Additional factors that might have led to a preference for temporal spiking elements over the course of evolution are related to network construction mechanisms. In [9] it was shown that in a fully connected cell assembly, where synaptic plasticity is time-dependent, a small number of neural clusters are formed, thus splitting the cell assembly into chained pools, and producing a distributed and synchronized firing pattern. This finding and others [10] show that a minimal temporal structure- based spiking activity can be learned in a self – organizing process.

In this study we examine whether temporal computing elements can emerge in small networks during evolution. It is based on evolutionary simulations of neurocontrolled virtual organisms that evolve in an environment with selective pressure for successful mate-finding. The virtual organism's reproduction model is based biological, genetic and neural development principles. The evolutionary simulations are based on a chromosome pattern that translates to a gene-protein network of a cellular organism controlled by a neural system. The chromosome model permits reproduction of an offspring by combining two chromosomes. During each evolutionary session selective pressure based on mate finding is placed on a population of neurocontrolled organisms. The results are based on the analysis of temporal neural coding in the evolved organisms.

Typically, in evolutionary simulation experiments a population of virtual organisms is evolved using a genetic algorithm [11] over many generations to best survive in a given environment. (See [12] for a full introduction), while there is full control of the environment and conditions, complete knowledge of the organisms' behavior, the network architecture, and dynamics. The present study is based on a complex, biologically plausible evolutionary model we presented elsewhere [13] that has been shown to evolve other unrelated biological phenomena such as gene order functionality [14]. Because of the important role mate finding and selection play in biological evolution [15], the data are taken from experiments in which the evolutionary pressure was based on mate finding and reproductive behavior.

Evolutionary models in neuroscience studies have been applied in a variety of ways: evolving a NN model of touch sensitivity behavior in *C.Elegans* [16]; studying the evolution and development of central pattern generators [17]–[19]; simulating the emergence of command neurons [20]; and in evolving “Mexican hat” patterns of activity [21].

Information theory was applied to find cases of evolutionary sessions in which there was a significant emergence of temporal neural coding in the evolved organisms. Our results suggest that such an emergence is repetitive and almost inevitable in some simulated environments.

## Results

In the next sections we first present our evolutionary simulation model that expands our previous constructs [13], [14] to include neural mechanisms. The first section describes our chromosome model that is based on DNA and protein-like sequences. Two such chromosomes can reproduce an offspring chromosome, as detailed in the second section. The translation model of chromosomes to gene-protein networks and the gene-network dynamic system model is detailed later, preceding sections describing the way the cellular dynamics is translated to organism and cellular functionality, differentiation and neural activity. After describing the model, we present evolutionary sessions where the virtual organism evolved in an environment with mate-finding selective pressure, and present various experiments and analyses examining the emergence of temporal neural coding in the evolved organisms.

### Chromosome

Each organism in the model expresses a phenotype derived from a chromosome structured according to biological foundations. Each chromosome includes a sequence of genes, where each gene starts with a promoter sequence followed by a messenger RNA sequence.

Each promoter sequence includes 1–3 cis-regulatory elements, and an element that includes the gene parameters. The parameter block of a gene/protein represents the properties derived specifically from its spatial structure. The use of gene and protein parameters in building the network is detailed later. A list of all such parameters is presented in Table 1. Each mRNA sequence starts with a cis-regulatory element, followed by a parameter sequence^1^, which in turn is followed by a trans-acting element; all represent the translated protein. All cis-regulatory elements, trans-acting elements and parameter sequences are represented as sequences of real numbers, with the chromosome composed of a long sequence of real numbers *r_1…_r_n_*. The chromosome is translated into a gene-protein network as detailed in the following sections.

**Table 1:** Gene/Protein parameters derived directly from the genome.

_θ_ *^a^*	Threshold value for gene/protein activation
θ*^p^*	Threshold value for protein production
β*^a^*	Slope value for gene/protein activation
β*^p^*	Slope value for protein production
α	Gene/Protein static activity factor
*k*	Protein diffusion factor
τ*^a^*	Gene/Protein activation time constant
τ*^p^*	Protein production time constant
**B**	A vector of two Boolean parameters that govern the translated protein's anchoring type on the membrane: i.e. whether the protein is anchored to the internal or external side of the membrane, or acts as a receptor that delivers information into or out of the cell.
***k_type_***	A vector of Boolean parameters that governs the translated protein's ability to diffuse between soma-axon, soma-dendrite, synapsed dendrite-axon.

The parameters above are encoded for each gene/protein in the chromosome as a “parameter block” and govern the gene and its derived protein dynamics in the gene-protein network. The model separates the activation dynamics, controlling the ability of the gene-protein to affect other genes-proteins, and the production dynamics that controls the protein's concentration, by having different slopes β^•^, thresholds θ^•^, and time constants τ^•^: β*^a^*, θ*^a^*, τ*^a^* for each gene/protein to control the dynamics of the activation and θ*^p^*, β*^p^*, τ*^p^* to control the dynamics of the protein production.

### Reproduction

A reproduction of a child chromosome from its parent chromosomes is based on a self adaptive method [22], avoiding linkage of the experimental results to specific crossover and mutation values. Each real value *r_i_* of the chromosome is surrounded by several other values: a crossover probability value *c_i_*, and two mutability values 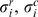 that control the extent to which parameters *r_i_* and *c_i_* respectively are likely to change (for more information see [22]). The values of 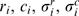 are mutated self-adaptively:
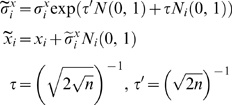
Where *n* is the number of genes, *x* ∈{*r*,*c*},*i* ∈{1‥*n*}, *N*(0,1) is a standard normal random number, *N_i_*(0,1) represents a new random number generated for each component, and 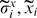 are the new values for 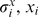.

Before mutation takes place, the parent chromosomes are aligned using a dynamic programming algorithm [23] and recombined where the probability for a crossover point to occur on the aligned chromosomes at location *i* & *j* of the parents is *P_ij_* = *c_i_*+*c_j_*.

### Gene-Protein network

The chromosome presented above is translated into a gene-protein network. The network connection strengths *w_ij_* are assigned according to the hamming distance *d_ij_* between cis-regulatory elements and trans-acting elements. Each gene and each protein transcripted has several parameters that are read from the chromosome and control its dynamics as detailed in Table 1.

The gene-protein network controls three dynamic values for each protein *i*: 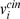 - The protein concentration inside the cell. 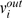 - The protein concentration outside the cell, and 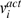 - the activity level of the protein in the cell. This value represents the extent to which the current spatial structure of the protein enables it to act on other genes and proteins.

The dynamics of the system is as follows:
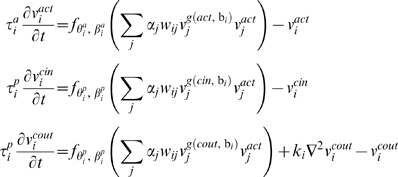
where 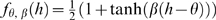, The equations above are based on a threshold logic paradigm commonly used in simulations of genetic regulatory circuits [24], [25], and neural networks [26], [27], where the basic differential equation is of the form:
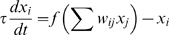



In such an equation the dynamics of a node value *x* are controlled with a time constant τ, and an activation function *f* that processes the cumulative field induced by the other nodes.

In the model, the field induced by a node *j* on node *i* is the product its dynamic activity level 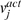, its concentration 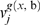, its concentration 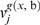, its static activity factor α*_j_*, and the connection between the nodes *w_ij_*.

To enable the model to separate the activation dynamics and the production dynamics, for example to affect a protein's concentration without affecting its spatial structure and vice versa, each gene/protein possesses different slopes β^•^, thresholds θ^•^, and time constants τ^•^: β*^a^*, θ*^a^*, τ*^a^* to control the dynamics of the activation and θ*^p^*, β*^p^*, τ*^p^* to control the dynamics of the protein production.

The model enables the external concentration 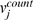 of each protein to play a role in the network dynamics by incorporating the expression 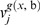 in the equations above. 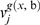 is either the internal 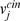 or external concentration 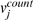, according to the values of x and b, which makes the model capable of evolving receptor-ligand relationships, based on the Boolean parameter b.

In order to permit tissue related dynamics, the external concentration equation contains a diffusion expression. *k_i_* is the diffusion coefficient of *i*, and 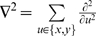, so that the expression 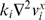 represents the contribution of diffusion to the change in external concentration, according to the diffusion equation 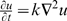.

The genetic aspects of the organism model are described more fully in [13], [14], [28].

### Cell functionality

In order to enable the gene-protein network presented above to model processes at the tissue level, we added output nodes to the gene-protein network. A similar component was introduced in [25] as a grammar of rules which describe inter- cell interactions and changes in number, type and state of cells. In our model, there is an output node *m* representing each cellular- related event that can be triggered by the network (apoptosis, mitosis, cellular migration, and differentiation, neurite sprouting, synaptic target selection), values that need to be derived from the network (like Na conductivity, synaptic weight regulation), or from the genome (such as translocation probability), including modeling directional receptors for axon guidance.

Each such output node *m* is represented by a random-generated bit string *s_m_*. The protein nodes *j* in the gene-protein network that are close enough to string 
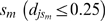
 are connected to output node *m*. According to the threshold logic paradigm mentioned earlier, an internal value *u_m_* is calculated for each output node:
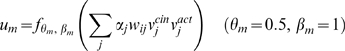



For nodes that trigger an event (e.g., occurrence of mitosis, cell death, migration, differentiation event), the event is triggered when the value *u_m_* passes a predefined threshold (0.5). When managing scalar values such as a translocation probability, the internal value *u_m_* may be multiplied by another pre-defined factor to obtain the actual scalar value as detailed in Table 2.

**Table 2:** List of all functions used in the experiments.

Description	Symbol	Output Type	Predefined Range
Cell differentiation messenger		B	{T,F}
Sensory neuron marker		A	(0,1)
Motor neuron marker		A	(0,1)
Hidden neuron marker		A	(0,1)
Mitosis messenger		B	{T,F}
Apoptosis messenger		B	{T,F}
Migration speed soma		A	(0,0.1)
Migration speed neurite		A	(0,0.1)
Sprout neurite messenger		B	{T,F}
Turn to adult messenger		B	{T,F}
Translocation Probability		A	(0,1)
Soma Migration Directional Marker		C	(0,*2*π)
Axon Migration Directional Marker		C	(0,*2*π)
Dendrite Migration Directional Marker		C	(0,*2*π)
Crossover Probability		A	(0,1)
Axon Target Select Marker		B	{T,F}
Synapse Weight Axon		A	(0,1)
Synapse Weight Dendrite		A	(0,1)
Inhibitory Neuron Marker		A	(0,1)
Odor A Sensor Marker		A	(0,1)
Odor B Sensor Marker		A	(0,1)
Sight Sensor Marker		A	(0,1)
Threshold potential	θ_0_	A	(−60E-3, −70E-3)
Threshold adaptivity factor	α	A	(0.005, 0.05)
Threshold time constant	τ_θ_	A	(15E-3,50E-3)
gNa in open channel state		A	(4.0,4.4)
gNa in closed channel state		A	(20E-3,50E-3)
Action Potential Refractory Time	τ*_ref_*	A	(2E-3,5E-3)
k Refractory Time	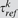	A	(4E-3,6E-3)
gk in open channel state		A	(200E-3, 500E-3)
gk in closed channel state		A	(2.3,2.6)
Synaptic current rise time	τ*_s_*_1_	A	(0.5E-3,2E-3)
Synaptic current decay time	τ*_s_*_2_	A	(3E-3,7E-3)
Membrane time constant at rest:	*C*/Σ*g*	A	(0.005, 0.02)
Photoreceptors sight angle	α*_pr_*	A	(0, π/*2*)
Neural noise time constant		A	(0, 1E-2)

The function values were limited to be in the ranges above. Type ‘A’ functions transform u_m_ linearly to be in a predefined (min,max) range. Type ‘B’ functions are Boolean functions based on a u_m_>0 test. Output Type ‘C’ functions are directional functions and are based on u_mx_ & u_my_ detailed earlier and produce an angle. All predefined ranges were chosen to cover reasonable biological values. Migration speed values are given in cell diameters per epoch. Neural electric properties are given in OASM like units, in the simulations each epoch represented half a millisecond. Only symbols of values that are referred to by symbol in the text are presented.

In cases a receptor-ligand relationship was needed to obtain directional quantification, a two dimensional version of the above value was used, where the effect of internal factors was replaced by the effect of external gradient factors:
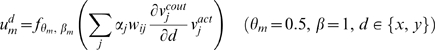



A list of all functions is detailed in Table 2.

In this paper the term ‘organism’ refers to the group of all cells that are repeated- mitosis results of the same zygote cell. Since during the mitosis the gene-protein network is copied from the parent cell, all organism cells are controlled by the same network structure, but since each cell is situated in a different location, it may possess different internal and external protein concentrations.

Each organism is allocated a period of time in which it must stop mitosis; only then will the organism be considered an adult that may reproduce. However, if the organism does not stop mitosis during the predefined period it is promptly removed from the environment without reproduction. This constraint is based on the assumption that an organism's ability to regulate its own growth and mitosis is a significant component of its fitness. We assume here that organisms that develop by infinite mitosis events are cancerous organisms that will suffer from low fitness values and therefore will not be able to reproduce [29].

### Cell Differentiation

When a cell differentiation messenger is triggered, the cell differentiates into one of three cell types according to its differentiation marker with the highest level (as detailed in Table 2):

- A motor cell – that upon firing will cause the agent to move in *l_m_-l_c_* direction, where *l_m_* is the motor cell location, and *l_c_* is the agent's centroid.
- A sensor cell – that will be either sensitive to an odorant (A or B), or act as a photoreceptor. Odor A is emitted into the environment by potential mate agents, odor B is emitted by non mate agents; the secreted current *I* from an odor sensitive cell is proportional to the distance from the odorant origin and the cell. Photoreceptor cells secrete constant current if any agent is placed in a pie region α*_pr_* radians wide.

A hidden cell – that will embed a neural model as detailed in the next section.

**Figure 1:**
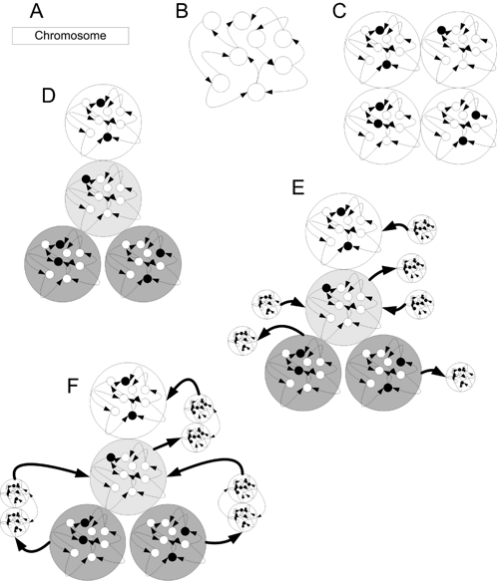
An example of development of a 4 cell agent. A) The development process begins with a chromosome. B) The chromosome is translated into a gene-protein network expressed in a zygote. C) The gene-protein network triggers mitosis events, producing 4 different cells. The network is the same in all cells, but the concentrations are different. D) The cells migrate and differentiate into a neuron, motor cell, and two sensory cells. E) Neurite sprouting events occur. Some proteins are marked by k_type_ as ones that cannot diffuse from neurite to soma. Therefore, their instances are separated in the neurites, with the same connectivity. F) After the axon is guided by external protein concentrations, target selection events occur, causing the axons to synapse. A synapse is formed, allowing proteins marked k_type_ as synapse-diffusible to move from one cell to another.

An example of the development of a 4- cell organism is illustrated in Figure 1.

### Neural activity

All hidden cells were embedded with an Integrate and Fire [30] neural model, where the membrane potential of the cell body behaved according to:
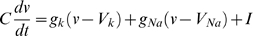
where *C* is the membrane capacitance, *I* is the total current injected into the cell, and *g* and *V* values are ion channel conductivity and reversal potential.

When the membrane potential reached the threshold θ, and the cell was not refractory, it fired an action potential, *g_Na_* was then raised for 1 system epoch, and immediately switched to a refractory state for τ*_ref_* seconds, where it could not fire and *g_k_* was raised and later decayed back with a 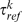 time constant.

The current *I* injected into the cell consisted of a noise current *I_noise_*, and incoming synapse current *I_exc_*, where *I_noise_* is a Gaussian noise causing a cell without external input to fire randomly. The noise of the various cells was uncorrelated.
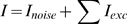



The excitatory *I_exc_* current injected by a presynaptic cell *i* into a postsynaptic cell *j* had a rise and decay time as follows:
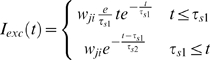
Where *t* is the time elapsed from the last action potential in the presynaptic cell, and τ_1_ & τ_2_ are the rise and decay time constants.

The threshold level had a dependence on the membrane potential level, according to:
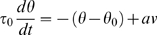



### The Evolutionary Session

The model presented above was used to simulate several evolutionary sessions in order to examine the temporal aspects of the evolved neural mechanisms. In order to make the evolved neural mechanisms biologically relevant, an effort was made to keep the model and the environmental definitions as unbiased and biologically plausible as possible. Each evolutionary session was initialized by placing a random population in an environment. Each agent was randomly set to be either male or female, and could move in the environment by using its sensor, motor and hidden neurons. In order to encourage the agents to develop neural networks, the agents were given a life span period proportional to the different cellular types they developed: a sensory neuron, a motor neuron, a hidden neuron, a dendrite, an axon, a synapse. Hence, a maximal time span was given to every agent that possessed a “basic” neural network, which was defined as a neural network with at least one instance of each of the six elements mentioned above. Since the system was defined as having only dendrite-to-axon synapses, a “basic” network could also be seen as a network that included at least a motor, a hidden and a sensory neuron and one synapse. The agents were removed from the environment after completing their life span period.

The population size was restricted to a predefined range by removing the eldest agents from the environment when the number of agents reached the upper bound (due to crowding), and by producing new individuals in the environment when the number of agents reached the lower bound.

In order to evolve neural based behavior, a “mating rule” was introduced in the environment, where two agents that contacted each other reproduced offspring according to the reproduction equation presented earlier. Accordingly, we expected the agents to develop neural mechanisms that would maximize their contacts with agents of the opposite sex.

**Figure 2:**
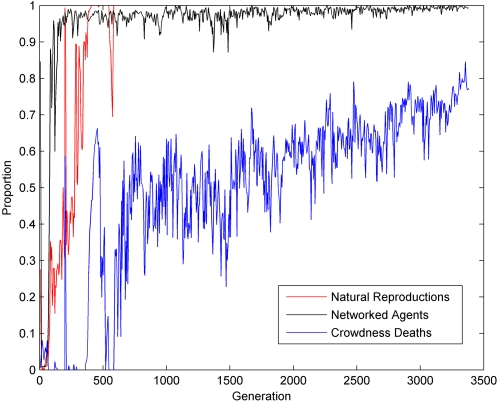
Behavioral development during evolution. Red: Proportion of reproduction triggered by agent contacts (as opposed to reproductions initiated by the system when the number of agents was too low). Black: Proportion of agents that developed a basic network (as defined in the text). Blue: Proportion of agent death events triggered by the system because of crowding (as opposed to deaths due to completing the life span period). The values are average proportions measured every 5 generations.

As a first step, we tested for changes in the agents' behavior along generations. As shown in Figure 2, the percentage of reproduction resulting from agent contact rose over generations. However, such a development could be a result of collective competence unrelated to individual neural mechanisms. In order to insure that this phenomenon was also based on individual competence, we saved the chromosome data from 200 randomly chosen agents during evolution, and tested each chromosome phenotype in a different environment that had two kinds of static objects: one with a “mate” odor, and the other with a “self” odor. The findings show a significant improvement over evolution in the average proportion of agent-mate contacts each 100 generations. (P = 1.80×10^−3^, r = 0.58, Spearman's Rank Correlation Test). Thus, during the evolutionary session there was some improvement in individual fitness.

### Static Mutual information

After demonstrating behavioral development in the evolutionary sessions, we tested for development in the neural representation.

Development in individual agents' ability during evolution to access the right objects implies that during evolution there may be some development in the neural representation of the environment that can be measured as an increase in the mutual information between the neural state of the agents and their proximal environment. An agent's fitness development can also be related to an increase in the agent's ability to exploit representational information for its activities.

**Figure 3:**
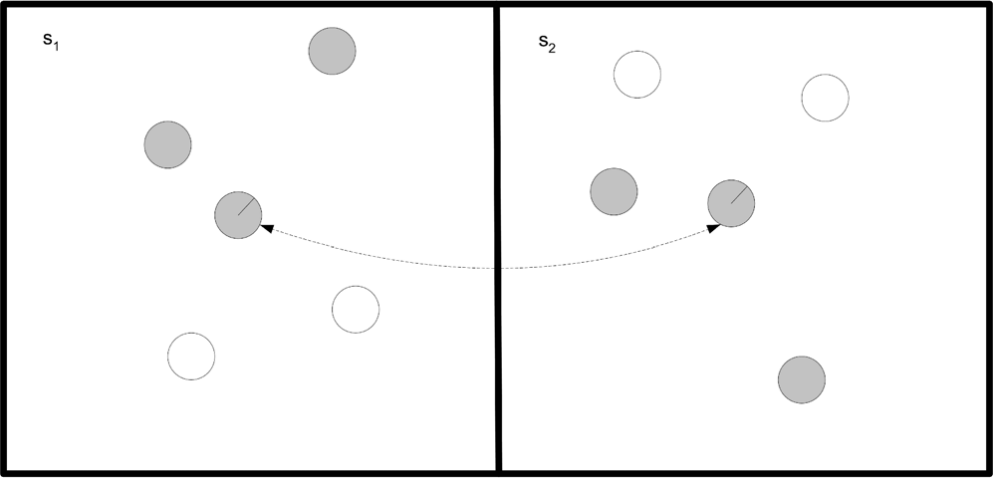
Static estimation of the mutual information between the agent's neural representation and the environment. The agent (arrowed) is moved between the center of s_1_ and s_2_ in a random order while its neural activity is recorded. The agent is pinned to the center of s_1_ and s_2_ and cannot move freely. s_1_ and s_2_ are two different environments containing agents of the same sex (grey) or opposite sex (white) at random locations.

To assess whether there was any development in the mutual information between the agent's environment and its neural representation we ran another evolutionary session where we saved chromosomes from randomly chosen agents during evolution. After the evolutionary session, the chromosomes were re-developed into agents, and a set *S* of two randomly generated environments was defined *S* = {*s*_1_, *s*_2_}, each *s_i_* having 4 static agents, two of each sex located randomly, as shown in Figure 3. The agents were pinned to the center of *s_1_* and *s_2_* repeatedly in a random order and their neural activity was measured. We calculated 4 mutual information measures for each agent using 4 different approaches:

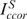
: The best estimated mutual information between the environment *S* and the cross correlation value of two neurons in the agent.

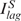
: The best estimated mutual information between the environment *S* and a time-lag value of two neurons in the agent.

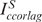
: The best estimated mutual information between the environment *S* and a combination of cross correlation and time lag of two neurons in the agent.

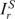
: The best estimated mutual information between the environment *S* and rate measures of two neurons in the agent.
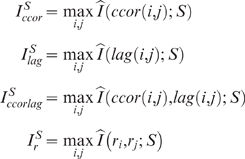

**Figure 4:**
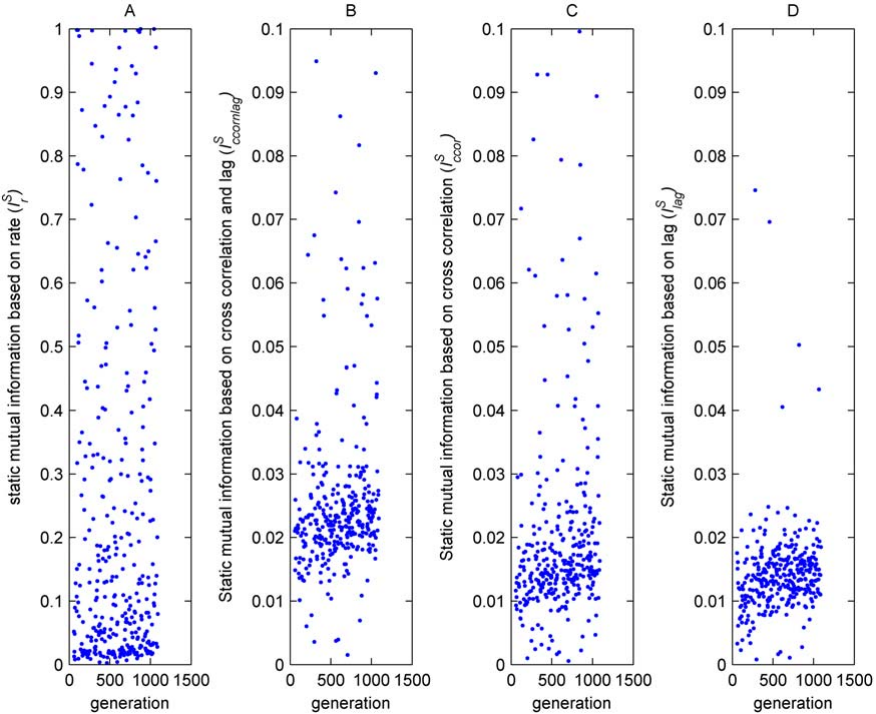
Best estimated mutual information with static environment values of randomly selected agents during evolution. A) The rate based measure 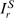 has higher values than the other measures, but no significant correlation was observed with generation (P =  0.7087, r =  2×10^−2^). B) Measure based on cross correlation combined with lag 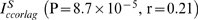. C) Measure based on cross correlation alone 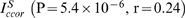. D) Measure based on lag alone 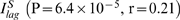. All values are based on Spearman's Rank Correlation Test made on 340 randomly chosen agents from the same evolutionary session. Please note the different axis in A.

As shown in Figure 4, there was a significant correlation between the current generation and each of the 3 time- dependent measures: 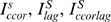.

However, such significance could not be found with the rate based measure 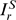. This could be attributed to a tendency of the evolutionary session to (i) ignore the rate based information. (ii) evolve systems that utilize the rate information without improving it. (iii) evolve systems that improve the rate based information which cannot be measured in static environmental conditions as presented earlier, but rely on a dynamic environment which is more comparable to the conditions where the agent has evolved. Accordingly, a dynamic experiment, which is described next, was designed to test the latter explanation by examining the growth in the mutual information between the neural representation and a dynamic definition of the agent's environment.

### Dynamic Mutual information

**Figure 5:**
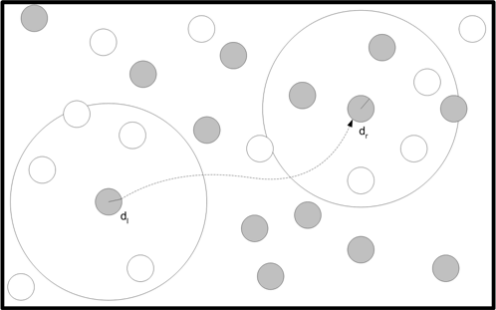
Dynamic estimation of the mutual information between the agent's neural representation and the environment. The agent (arrowed) moved in a single environment freely. The external environment was defined as d_l_ or d_r_ when there were more agents of the opposite sex on its left or right respectively.

In this experiment, after the evolutionary session, the chromosomes were re-developed into agents, and put one at a time in a single environment similar to the one they evolved in, with two types of objects: a ‘mate’- like and ‘non- mate’ like object. The agents could move in the environment freely and their neural activity was assessed. A a “preferred direction” value *D* = {*d_r_*, *d_l_*} was continuously calculated for the agent, indicating whether there were more mate-like objects to its right (*r*) or left (*l*) (see figure 5). Four additional mutual information measures were obtained for each agent:

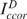
: The best estimated mutual information between the preferred direction *D* and the cross correlation value of two neurons in the agent.

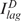
: The best estimated mutual information between the preferred direction *D* and a time-lag value of two neurons in the agent.

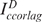
: The best estimated mutual information between the preferred direction *D* and a combination of cross correlation and time lag of two neurons in the agent.

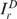
: The best estimated mutual information between the preferred direction *D* and rate measures of two neurons in the agent.
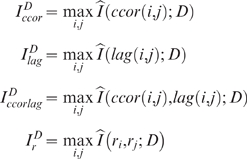

**Figure 6:**
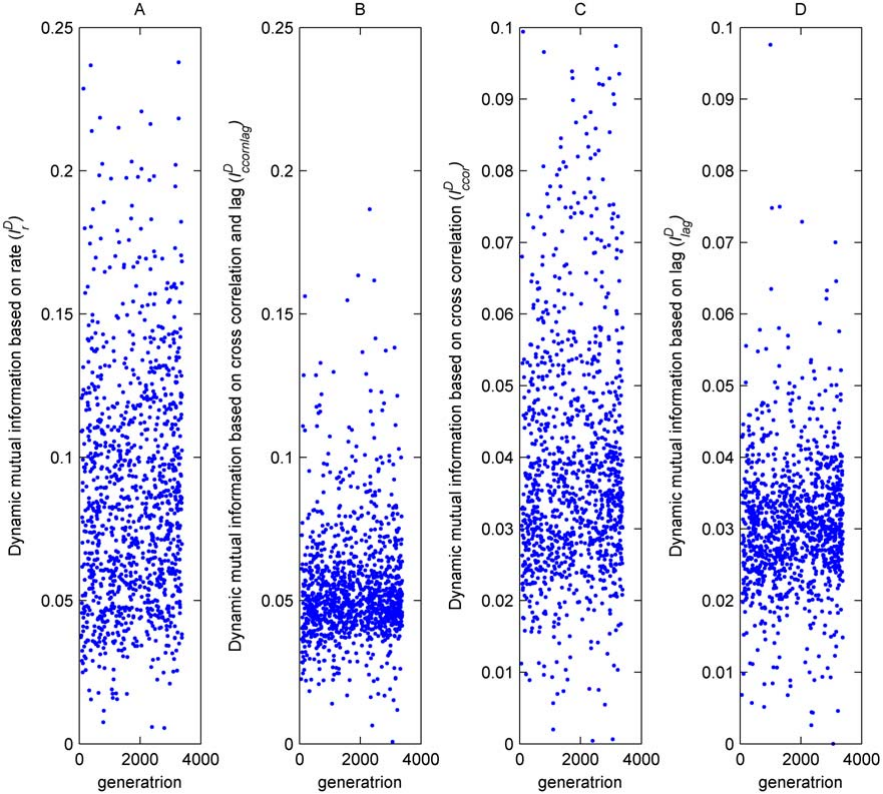
Best estimated mutual information with dynamic environment values of randomly selected agents during evolution. A) The rate based measure 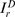 has higher values than the other measures, with a significant correlation 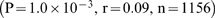. B) Measure based on cross correlation combined with lag 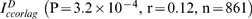. C) Measure based on cross correlation alone 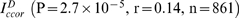. D) Measure based on lag alone 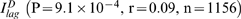. All values are based on Spearman's Rank Correlation Test made on randomly chosen agents from the same evolutionary session. Since the cross correlation based measures 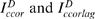 reached a plateau earlier than the others, their statistical tests were based on the first 2700 generations whereas the other tests were based on the entire evolutionary session. Please note the different axis in A and B.

As shown in Figure 6, there was a significant correlation between the current generation and all the dynamic mutual information measures:

### Utilizing the information

Although we showed there was significant growth of mutual information in the experiments above, in the time dependent cases, the absolute mutual information values were very small (as seen in Figures 4 and 6), suggesting that there was no evolutionary pressure to raise the mutual information values we have chosen to examine, and implying that the shown growth is not due to evolutionary dynamics of the model. Such a conclusion would also derive that there is no effect of the mutual information measures on the agents' fitness. In the next experiment we tested whether in these cases the information had any effect on the agents' performance; namely, whether there was any correlation between the mutual information in the previous analysis and actual performance. Again, the chromosomes were re-developed into agents, and placed one at a time in an environment similar to the previous one. The agents could move in the environment freely as their neural activity was measured. Additionally, in this test we also calculated *grade*:i.e., the proportion of contacts each agent had with a ‘mate’ object, divided by the ‘mate’ object frequency. We calculated the Spearman rank correlations between the mutual information measure and the grades of 1156 randomly selected agents. A significant correlation was only found for 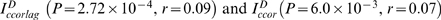. In the other cases the results were not significant: 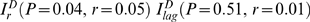. One possible explanation is that in this particular fitness landscape, the development of these information systems was simply a side- effect of the cross correlation in mutual information.

### Neural ablation

This latter finding raised the possibility that the increase in the major estimated mutual information measures such as 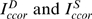 was actually a side effect of some other mechanism which was not directly related to precise firing time. By extension, the effect of ablating precise time neurotransmission abilities should have the same effect over evolution.

Therefore, to test the null hypothesis, we defined an ablated setting where the excitatory current injected by a presynaptic cell *i* into a postsynaptic cell *j* was changed to 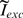 instead of *I_exc_* where:
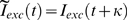
κ is a delay time randomly generated upon synapse creation: κ = *U*(0,1)*τ*_s_*_1_ and *U(0,1)* is a uniformly distributed random number between 0 and 1. Each agent was given two performance grades based the proportion of contacts each had with a ‘mate’ object, divided by the ‘mate’ object frequency:

*g*: Grade in regular *I_exc_* definitions.

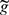
: Grade in a test with ablated current injection times based on 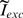.

**Figure 7:**
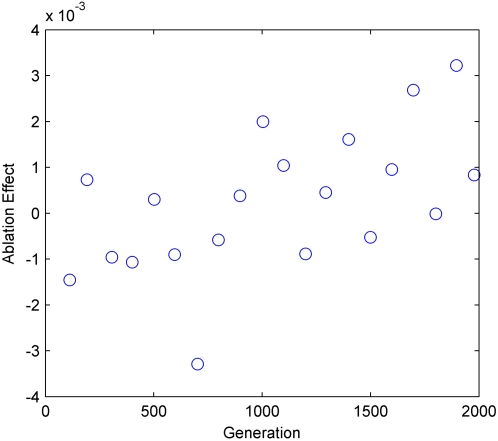
Ablation Effect correlated with generations. Each sample is the average ablation effect measured for a population of 100 agents in 100 generation bins. In each generation one agent was chosen randomly for this experiment. (P = 5.0×10^−3^, r = 0.63, N = 20, Spearman's Rank Correlation Test).

As shown in Figure 7, the ablation effect 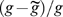 was indeed correlated with evolution.

It could be argued that this finding was unique to a specific evolutionary session. However after running 10 different evolutionary sessions with the model, in all evolutionary sessions a significant correlation was found (Max P<0.05, Spearman's Rank Correlation Test) between the ablation effect 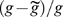 and the generations.

## Discussion

It has been suggested that some biological structures may be evolutionary inevitable in a given environment [31]. Numerous studies have also reported precise time relations among spikes [32]–[35]. Our results combine these two findings and show that in some evolutionary models, such as the one presented here, evolvement of precise time relations among spikes is almost inevitable.

In our preliminary experiments our null hypothesis was that different information measures based on precise time relations among spikes would not increase during evolution. We were able to show that this null hypotheses could be rejected both in static and dynamic environments in the conditions defined by our model. We also showed a connection between the cross correlation based measure and the fitness of the agents, suggesting that the increase shown previously was due to evolutionary forces. Subsequently, we showed that ablation of precise time neurotransmission abilities has an increasing effect over evolution in each independent evolutionary session of our model.

Although both dynamic and static experiments showed a significant increase in the time dependent measures, the different rate based measure results highlight the importance of environmental coding, (which in our case was dynamic *{d_l_,d_r_}* vs. static *{s_1_,s_2_}*), and not only the importance of the neural coding, (which in our case was *ccor* vs *ccorlag* vs *lag* vs *r*). We believe this feature has been unjustly overlooked in electrophysiological studies examining the mutual information between the external environment and neural code. Even in the mutual information experiments presented here, we believe much more significant results could be obtained after finding an optimal environmental code instead of querying simply whether there are more mates on the right or left side of the agent.

The results raise questions as to why the evolutionary sessions appeared to prefer basing the agents' dynamics on spike timing and not only on rate components. This could be a result of the dominance of a neural solution that is also based on spike timing or a biological infrastructure that enables faster convergence to such a solution. However, some trivial evolutionary mechanisms or experimental artifacts could also generate such a development. These include the following: (i) definition of the environment in which the evolutionary session took place as one where small time scale reactions are a significant component of the agent's fitness. (ii) mutual information growth that is a by- product of other processes and does not contribute to individual fitness directly. (iii) a coincidental evolutionary case that has no implications regarding the general evolutionary landscape. By considering the τ*_m_* value presented in our experiments we avoid definitions that could lead to the first case. Our latter experimental results disconfirm the second case. The third case does not seem possible in the light of repeated results in the experiments from different evolutionary sessions.

It should be noted that in this study the results are inferred from the average population values or from values obtained from randomly selected individuals. A more detailed investigation should be based on larger samples of particular phylogenies, especially to provide estimates of the population variance during evolution. The results presented here do not address the question of whether the population is homogeneous or whether there are only a few very successful individuals in the population yielding a greater fitness average.

Naturally, the relevance of the results in terms of biology is based on the applicability of the model. Although the model used is complex, we have tried to avoid pinning its parameters to certain predefined values, and most of the model parameters are self adaptive (see Table 2 & 3), making the conditions defined by the model biologically plausible. It is also likely that other, unrelated biological results found by a previous version of the model [14] contribute to the model's biological applicability. However, further research should be conducted by simplifying the model and deriving the essential model components that contribute to the development of time dependent neural elements.

**Table 3:** Range limits of the different chromosome parameters in the experiments.

θ*^a^*, θ*^p^*	Threshold values were limited to a range of (*−2n,+2n*) where *n* is the number of genes in the chromosome. This is due to the possible range the field value 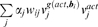 can have.
β*^a^*, β*^p^*	Slope values were limited to (0,10), assuming greater slopes to have same results as β = 10
α	(−1,1)
*k*	Were limited to (0,1),
τ*^a^*, τ*^p^*	The lower limit for all time constants was the time represented by a single epoch of the system; the upper limit was the maximal possible life period assigned to an organism.
*c*	Since the actual crossover probability is the sum of two crossover probabilities, crossover probability values were limited to (0,0.5).

The chromosome parameters were limited to have physically reasonable values as detailed above. The *c* value mentioned in the table is not part of a parameter block, but a chromosome related value in the model controlling the crossover probability, as detailed in the reproduction section.

The above findings, highlighting the importance of spike time precision from an evolutionary point of view, raise several questions concerning their generative source, the way the time precision is read out, and the causes that make this computational element so frequent in the model. Answering these questions using a simulative evolutionary model like the one presented here should be easier than answering them in the broad biological scope, and might shed some light on the structure of biological neural systems.

## Materials and Methods

### Calculating Cross Correlation values

The cross correlation *ccor* per time lag *d* series of two neurons *x* & *y* was calculated as follows:
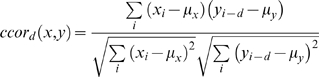
where *x_i_* is 1 if neuron *x* fires at time *i* and 0 otherwise. *y_j_* is 1 if neuron *y* fires at time *j* and 0 otherwise. μ*_x_* and μ*_y_* are the average values of *x* and *y* respectively.

The cross correlation of two neurons *ccor(x,y)* and the lag correlation *lag(x,y)* between them were calculated as follows:
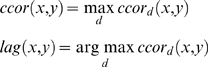



### Estimating mutual information

The estimation of mutual information of two stationary signal pairs is based on a biased histogram-based method to estimate mutual information as detailed in [36]. The information logarithm base is 2 (bits).

### Motor Cells & Movements

The effect of a motor cell was generated only τ*_m_U*(0,1) milliseconds after the motor cell fired. The correlation measures were made only in a τ*_m_* millisecond window. In the experiments a value of *25 ms* was used as τ*_m_*.

### Chromosome model

We used cis and trans elements as sequences of 16 real numbers. Several evolutionary simulations were run with different cis and trans lengths (8 or 32 numbers); a significant correlation for these lengths was also found between 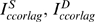 and the generation *(P<0.05)*. As “parameter blocks” we used sequence numbers representing the parameters in Table 1. In order to keep these values within reasonable ranges, the values were limited to predefined ranges as detailed in Table 3.

### Population size

The population size was forced to be in the range of 100±10 simultaneous agents by removing agents or producing new agents when the number of agents reached or exceeded the population size limits. Agents were also removed from the environment after passing their fitness-based life span and added to the environment when their parents contacted each other. Therefore, successive generations could overlap.

Several sessions with a different population size of 500 were examined regarding the correlation between the generation and the 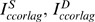 values, without observing a significant change in the results (Maximal P<0.05).
